# Supplementary material for: Investigation of the Low-Populated Excited States of the HIV-1 Nucleocapsid Domain
Source: Viruses. 2022 Mar 18;14(3):632. doi: 10.3390/v14030632 (PMC8950434; doi:10.3390/v14030632)
Supplement: Supplementary file 1 [file viruses-14-00632-s001.zip › viruses-1617511-supplementary.pdf]

	$CEST_{H44,W37,C39}$+ $CPMG_{H44,W37,C39}$	$CEST_{H44,W37,C39}$+ $CPMG_{10residues}$	$CPMG_{10residues}$ $R2_{950MHZ}$	$CEST_{H44,W37,C39}$	$CEST_{H44,W37,C39,G40,E42}$+ $CPMG_{H44,W37,C39}$
**W37 R2_A (s^{-1})**	5	5	7.7	5.00	5
**W37 R2_B (s^{-1})**	100	100	7.7	99	100
**W37 R2_C (s^{-1})**	5	5	7.7	5	5
**C39 R2_A (s^{-1})**	5	5	7.4	5	5
**C39 R2_B (s^{-1})**	100	100	7.4	100	100
**C39 R2_C (s^{-1})**	5	5	7.4	5	5
**H44 R2_A (s^{-1})**	5	5	6.4	5	5
**H44 R2_B (s^{-1})**	100	100	6.4	67	100
**H44 R2_C (s^{-1})**	100	100	6.4	100	99

Supplementary Table S1. ^{15}N transverse relaxation rates (R2) for the three different states obtained from the fit of CPMG data or CEST data for NCp7 measured at 35°C with ChemEx and for three residues (W37, C39, H44). In the three-state model, A is the major form and B and C are the minor forms with mutual interconversion between the three states. In these calculations, different sets of data have been used as described in Table2. Note that the bounds for R2 rates were fixed to (5,100), these bounds are always reached at the end of the calculations.

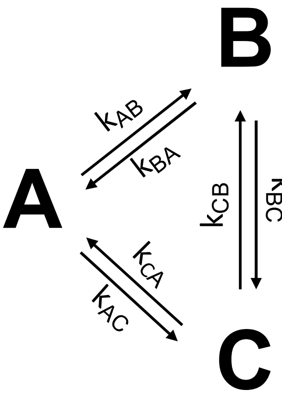

Supplementary Figure S1. Three-state model between a native or major state A and two-sparsely populated states B and C.

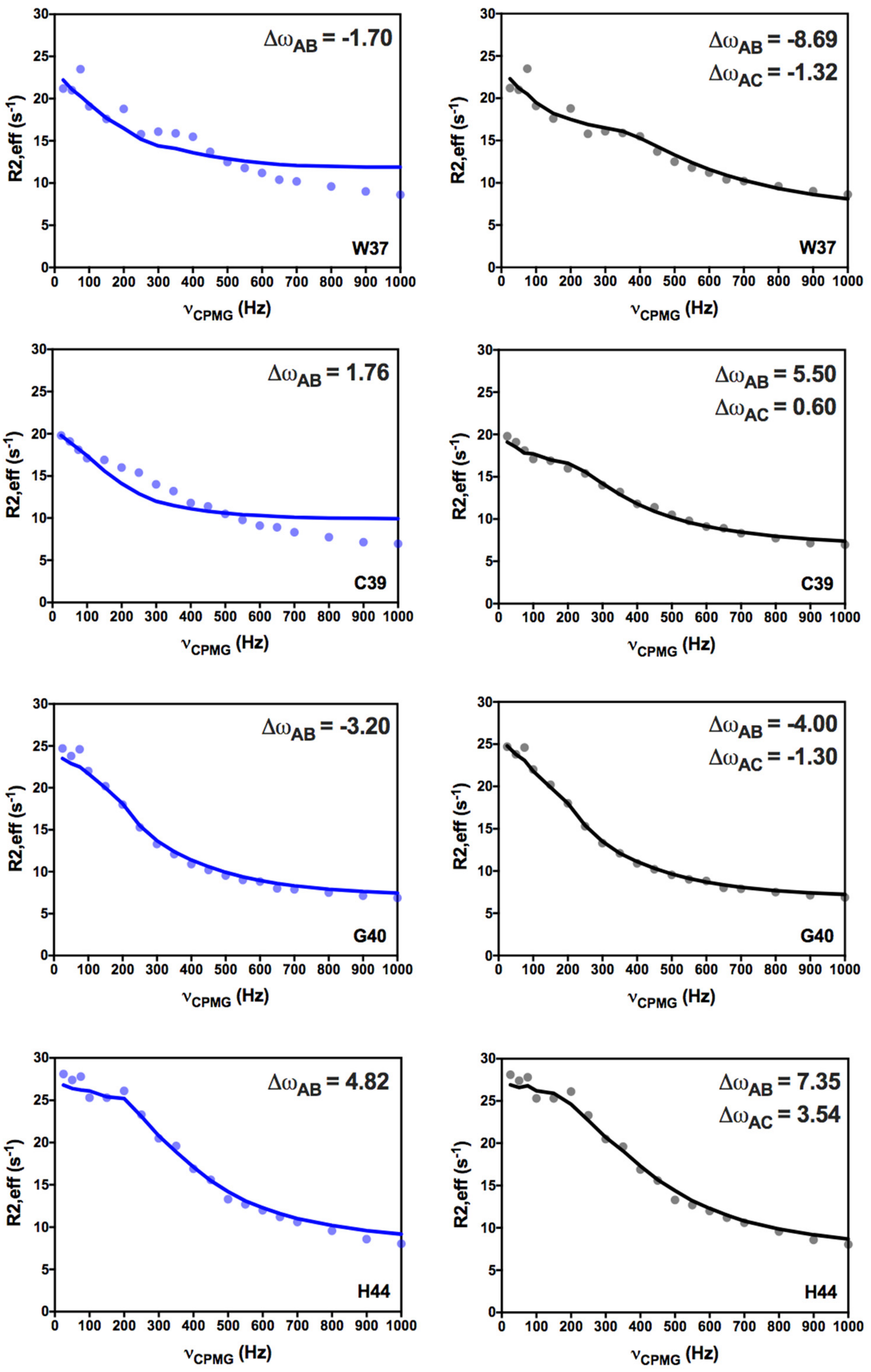

Supplementary Figure S2. ^{15}N-CPMG relaxation dispersion profiles for residues W37, C39, G40 and H44 of NCp7 at 700 MHz and 35°C. The experimental data (dots) have been fitted with ChemEx using either a two-state model (left, blue line) or a three-state model (right, in black). For each residue and each model, the chemical shift values $\Delta\omega_{AB}$ and/or $\Delta\omega_{AC}$ are indicated in ppm.
